# Predicting Low and Non-Responders and Outliers in Patients with Spinal Cord Injury

**DOI:** 10.3390/jcm15114167

**Published:** 2026-05-28

**Authors:** Giorgio Scivoletto, Emanuela Lena, Laura Barrucci, Valeria Di Pasquale, Simone Tiberti, Serena Vincenza Capobianco, Calogero Foti, Luisa Maria Lapenna, Stefano Filippo Castiglia, Federica Tamburella

**Affiliations:** 1 Unità Spinale Unipolare, Ospedale CTO “Andrea Alesini”, 00145 Rome, Italy; simone.tiberti@aslroma2.it (S.T.); serenavincenza.capobianoc@aslroma2.it (S.V.C.); 2 Spinal Center, IRCCS Fondazione S. Lucia, 00179 Rome, Italy; e.lena@hasantalucia.it (E.L.); v.dipasquale@hsantalucia.it (V.D.P.); 3 Spinal Rehabilitation (SpiRe) Lab, IRCCS Fondazione S. Lucia, 00179 Rome, Italy; f.tamburella@hsantalucia.it; 4 Specialization School of Physical Medicine and Rehabilitation, Tor Vergata University, 00133 Rome, Italy; barrucci8laura@gmail.com (L.B.); foti@med.uniroma2.it (C.F.); 5 UOS of Physical Medicine and Rehabilitation, Ospedale Generale Cristo Re, 00167 Rome, Italy; luisamaria.lapenna@ospedalecristore.it; 6 Laboratory of Neuromotor Physiology, IRCCS Santa Lucia Foundation, 00179 Rome, Italy; stefanofilippo.castiglia@uniroma1.it; 7 Department of Medico-Surgical Sciences and Biotechnologies, “Sapienza” University of Rome, Polo Pontino, 04100 Latina, Italy; 8 Department of Life Sciences, Health and Health Professions, Link Campus University, 00165 Rome, Italy

**Keywords:** spinal cord injury, outliers, outcomes, length of stay, discharge destination

## Abstract

**Background:** Patients with spinal cord injury (SCI) typically have longer lengths of stay (LOS) compared to other rehabilitation patients and show variable therapeutic responses. Identifying reliable predictors of functional outcomes is essential. This study aimed to find prognostic factors to detect patients with high/low functional response, prolonged LOS, and those discharged home. **Methods:** We retrospectively reviewed charts of SCI patients admitted to our center since 1997, recording neurological status (International Standards for Neurological Classification of SCI), Spinal Cord Independence Measure (SCIM), and Walking Index for SCI (WISCI). Rehabilitation results were measured by treatment effectiveness, reflecting the proportion of potential improvement achieved. Patients with SCIM and WISCI effectiveness above the mean plus one standard deviation (SD) were considered outliers, as were those with LOS exceeding the mean plus one SD. Forward stepwise logistic regression identified variables linked to high effectiveness, LOS, and discharge destination. **Results:** A total of 1059 patients were included (739 males, mean age 50.8 ± 18; 587 with non-traumatic etiology). There were 132 LOS outliers, 163 high SCIM responders, and 144 high WISCI responders; 913 were discharged home. Regression analysis found that year of admission, older age, and complications at admission and during rehabilitation were independently associated with reduced likelihood of high SCIM response; complications at admission and during rehabilitation were associated with reduced likelihood of high WISCI response; year of admission, older age, longer lesion-to-admission time, and complications during rehabilitation predicted prolonged LOS; and more recent year of admission, traumatic etiology, and lower discharge SCIM were associated with nursing home placement. **Conclusions:** Our data provide a basis for further research into the problem of SCI prognosis. Subgroups of subjects with poor or excellent rehabilitation prognosis could be recognized at the beginning of treatment based on clinical factors.

## 1. Introduction

Spinal cord injuries (SCI) and lesions occur less frequently than other disabling conditions, such as stroke. The estimated incidence of traumatic SCI ranges from 3.3 to 195.4 cases per million people per year, depending on the country; however, obtaining reliable data remains challenging in some regions [1]. Information on non-traumatic SCI is even more limited and often of lower quality. Due to methodological issues—including inconsistent inclusion and exclusion criteria, incomplete diagnoses, and underreporting—the reported incidence of non-traumatic SCI varies widely, from 11.4 new cases per million in Spain to 68 per million in Canada [2].

Despite their lower incidence, SCIs are associated with substantial economic burdens during and after inpatient rehabilitation [3]. Because SCI affects multiple functional domains, rehabilitation must be multidisciplinary, incorporating motor therapy, occupational therapy, respiratory training, urological rehabilitation, and, when needed, speech and swallowing therapy. The cost of inpatient rehabilitation varies considerably, averaging about $106,890 in Canada and $190,620 in the United States, and can reach up to $433,044 depending on lesion level and severity, associated injuries, and complications arising during the rehabilitation period [3].

Although inpatient SCI rehabilitation is both costly and time-consuming, it has been shown to improve functional outcomes and enhance quality of life [4]. Nevertheless, SCI patients generally experience longer lengths of stay (LOS) than other rehabilitation populations [5] and show heterogeneous responses to therapy. A large intermediate group demonstrates neither clearly favorable nor clearly poor prognoses, while smaller subgroups exhibit distinctly excellent or poor outcomes [5]. This variability highlights the need for reliable predictors of functional recovery. Accurate outcome prediction is essential not only to avoid unnecessary occupancy of rehabilitation beds but also to establish realistic therapeutic goals, allocate resources efficiently, and anticipate individual patient trajectories [6].

Furthermore, until recently, the pathogenesis and progression of spinal cord damage were not fully understood [7]. Current knowledge has expanded, particularly regarding the roles of inflammation and vascular mechanisms in SCI. As a result, several neuroprotective strategies—especially those involving anti-inflammatory agents—have been proposed and tested in clinical trials [8]. In addition, various neuro-regenerative and rehabilitative interventions are employed to improve neurological and functional outcomes in individuals with SCI. To properly assess the effectiveness of these approaches, a precise understanding of the natural history of SCI and the factors influencing its progression is essential.

Based on these considerations, this study had the objective of identifying reliable admission-based prognostic factors capable of classifying patients into subgroups characterized by no, low, or high response to rehabilitation, prolonged LOS, and likelihood of discharge to home among individuals admitted for first-time SCI rehabilitation.

## 2. Materials and Methods

We conducted a retrospective review of the medical records of SCI patients admitted to our facilities from 1997 onward.

Inclusion criteria:

- Newly occurring traumatic or non-traumatic SCI;
- Age > 18 years;
- Cognitive ability sufficient to participate in rehabilitation.

Exclusion criteria:

- Pre-existing neurological diseases or trauma;
- Evidence of disease progression;
- Readmissions after discharge or transfer periods longer than three weeks;
- Hospital stays shorter than seven days;
- Incomplete clinical data.

For all patients, the following information was collected:

- Year of admission.
- Sex and age.
- Etiology (traumatic or non-traumatic).
- Complications at admission and arising during rehabilitation. The following were classified as complications: pressure ulcers, deep vein thrombosis, pulmonary embolism, heterotopic ossification, and urological complications (excluding urinary tract infections).
- Presence of lesions associated with the spinal cord lesion (SCL) in patients with traumatic injuries. These associated lesions included traumatic brain injury, non-vertebral fractures requiring surgery, severe facial trauma involving sensory organs, major chest injuries requiring chest tube placement or mechanical ventilation, severe hemorrhage, and internal organ injuries requiring surgical intervention.
- Level of independence in bladder and bowel management at discharge.
- Length of rehabilitation stay (LOS).
- Discharge destination (0 = home discharge, 1 = discharge to a nursing facility).
- Neurological status assessed according to the International Standards for Neurological Classification of Spinal Cord Injury [9]. This assessment includes motor testing of 10 key muscles on each side of the body, yielding the total Motor Score as well as the Upper Extremity Motor Score (UEMS) and Lower Extremity Motor Score (LEMS). Light-touch (LT) and pin-prick (PP) sensation were evaluated across all dermatomes. Based on these findings, lesion severity was classified into five categories according to the American Spinal Injury Association Impairment Scale (AIS), ranging from AIS A (complete lesion with no sensory or motor function in the most caudal sacral segment, S4/5) to AIS E (normal sensory and motor function).
- Spinal Cord Independence Measure (SCIM), version II or III [10], to assess functional status at admission and discharge. The SCIM includes three subscales: self-care (0–20), covering feeding, bathing, dressing, and grooming; respiration and sphincter management (0–40), including respiratory function and bladder/bowel management; and mobility (0–40), covering bed mobility, transfers, indoor/outdoor mobility, and stair climbing. Total scores range from 0 to 100, with higher scores indicating greater independence.
- Walking Index for Spinal Cord Injury II (WISCI II) [11] to evaluate walking ability. This 20-point scale rates ambulation based on the need for physical assistance, braces, and assistive devices.

The rehabilitation program consisted of individualized physiotherapy sessions lasting 60 min, delivered twice daily (with one session on Saturdays) for six days per week. When indicated, patients also received respiratory training or speech and swallowing therapy. Occupational therapy was provided two to three times per week to improve performance in activities of daily living. Both physiotherapy and occupational therapy continued throughout the entire hospitalization.

Patients with excellent or poor prognoses in terms of ADL and mobility were identified based on their deviation from the mean treatment effectiveness. This classification relied on the assumption that the range of mean ± 1 standard deviation (SD) includes approximately two-thirds of observations. Treatment effectiveness was calculated as [4,12]:

Effectiveness = [(discharge score − initial score)/(maximum possible score − initial score)] × 100

This metric reflects the percentage of potential improvement achieved during rehabilitation; thus, a patient reaching the maximum score at discharge attains 100% effectiveness. Effectiveness was calculated for both SCIM and WISCI scores.

Patients whose effectiveness fell within ±1 SD of the mean were classified as standard responders. Those with values beyond ±1 SD were categorized as excellent responders (high response group) or poor responders. The poor response group was further divided into “no response” (effectiveness = 0) and “low response” (effectiveness below mean − 1 SD but >0) [12]. LOS outliers were defined as patients whose rehabilitation stay exceeded the mean plus 1 SD [4]. Discharge destination was classified as either discharge home or transfer to a nursing home.

To control for the influence of lesion level and injury completeness, mean effectiveness and LOS were calculated separately for cervical, thoracic, and lumbar lesions, as well as for AIS A/B versus C/D groups. SCIM and WISCI outliers were identified based on their deviation from the mean effectiveness within these subgroups. The choice of defining outliers as those with an effectiveness or LOS who exceeded the mean plus 1 SD is an operational one and was chosen to remain consistent with previous rehabilitation “outlier” studies [12] and with the exploratory aim of identifying patients at the extreme end of the distribution.

The study was conducted in accordance with the Declaration of Helsinki and approved by the Ethics Committee of the Santa Lucia Foundation (protocol code CE/PROG.884, approval date 15 December 2020).

### Data Analysis and Statistics

Continuous and ordinal variables were summarized using means and standard deviations, whereas binary and nominal variables were reported as percentage frequencies. As most of the variables were dichotomous, logistic regression (forward stepwise) was used as an exploratory analysis to identify independent variables associated with high, low, or no effectiveness, high LOS, and discharge destination [4,12]. Independent variables included year of admission, sex (female = 0, male = 1), age, etiology (non-traumatic = 0, traumatic = 1), associated lesions (absent= 0, present = 1), and complications at admission or during rehabilitation (absent = 0, present = 1). Lesion level (cervical, thoracic, lumbar) and AIS grade (A/B vs. C/D) were not included as regression predictors, as outlier status had already been defined within subgroups stratified by these variables; entering them as covariates would have adjusted for their effect twice. For LOS and discharge destination, SCIM at discharge and independence in bladder and bowel management (independent = 0, dependent = 1) were also considered as candidate predictors. Furthermore, as a sensitivity analysis, we performed the same analysis, including the presence of pressure ulcers instead of the whole group of complications. Before performing the logistic regression, we assessed multicollinearity among candidate predictors. All the statistics were performed by means of SPSS version 20(^®^) (Chicago, IL, USA: SPSS Inc.). Microsoft 365 Copilot was used during manuscript revision to enhance readability and language clarity. All authors subsequently reviewed and edited the text, taking full responsi-bility for the accuracy and integrity of the final publication.

## 3. Results

**Figure 1:**
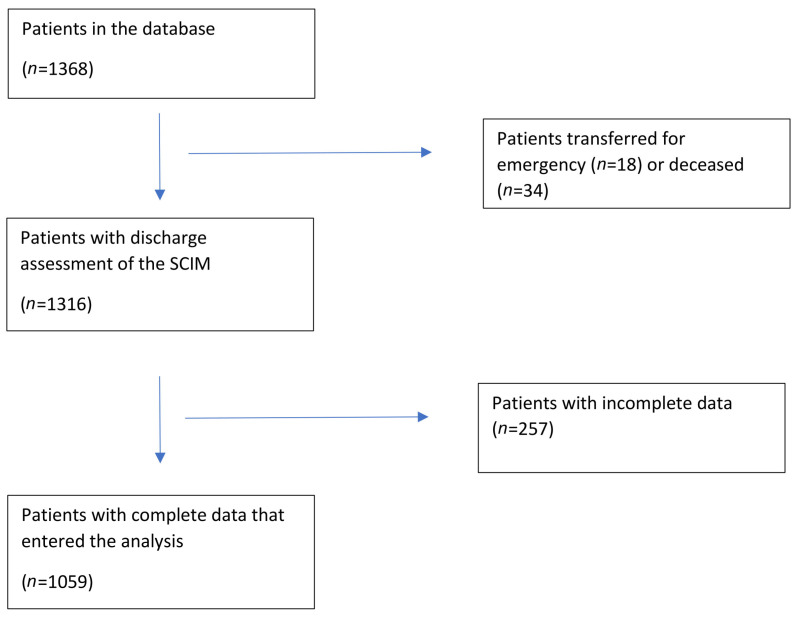
Flowchart of the patients inclusion/exclusion.

Of the 1368 patients assessed, 1059 with complete data were included in the final analysis (739 men; mean age 50.8 ± 18 years; 587 with non-traumatic etiology) (see Figure 1). Therefore, this is a complete-case analysis. In terms of neurological status, 93 patients had a cervical A/B lesion, 265 a cervical C/D lesion, 254 a thoracic A/B lesion, 243 a thoracic C/D lesion, 60 a lumbar A/B lesion, and 144 a lumbar C/D lesion. At admission, 267 patients already presented with complications—primarily pressure ulcers—while 318 developed complications during their rehabilitation stay. The mean length of stay (LOS) was 137 days.

The multicollinearity analysis showed good values of Variance Inflation Factor (VIF) and Tolerance (Supplemental Material, Table S1).

The logistic regression analysis identified significant association between several clinical and demographic variables and the outcomes under examination (SCIM and WISCI, length of stay and destination at discharge). For all the models analyzed, the Omnibus test was statistically significant (*p* < 0.001).

**Figure 2:**
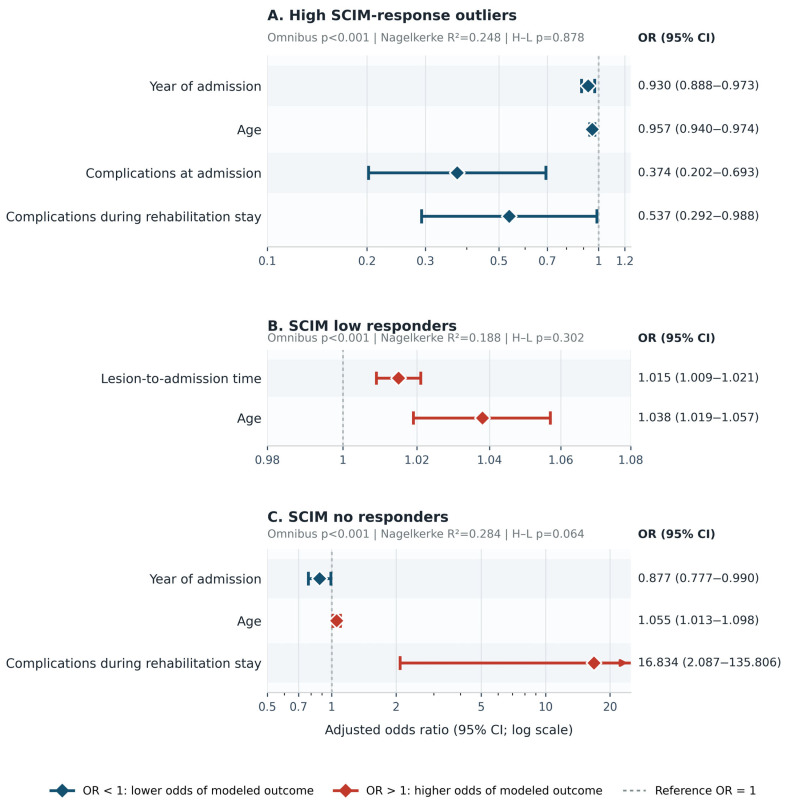
Adjusted odds ratios for predictors retained in the logistic-regression models for SCIM outcomes. Forest plots show adjusted odds ratios (ORs) and 95% confidence intervals (CIs) from forward stepwise logistic-regression models for: (**A**) SCIM high-response outliers, defined as patients with SCIM treatment effectiveness greater than the subgroup mean + 1 SD; (**B**) SCIM low responders, defined as patients with SCIM treatment effectiveness below the subgroup mean − 1 SD but greater than zero; and (**C**) SCIM non-responders, defined as patients with no SCIM improvement during rehabilitation. The dashed vertical line indicates OR = 1. ORs for continuous predictors are expressed per one-unit increase: one calendar year for year of admission, one year for age, and one day for lesion-to-admission time. ORs for complications are expressed as presence versus absence. Values below 1 indicate lower odds of the modeled outcome, and values above 1 indicate higher odds of the modeled outcome. All models included 1059 patients. SCIM, Spinal Cord Independence Measure; OR, odds ratio; CI, confidence interval; H–L, Hosmer–Lemeshow test.

Regarding independence in activities of daily living (as evaluated with the Spinal Cord Independence Measure), a total of 163 subjects showed a high SCIM response, while 45 patients were classified as SCIM non-responders. The likelihood of being a SCIM outlier is negatively associated with several variables: the year of admission (*p* = 0.002, Exp(B) = 0.930), age (*p* < 0.001, Exp(B) = 0.957), complications at admission (*p* = 0.002, Exp(B) = 0.374), and complications during rehabilitation stay (*p* = 0.046, Exp(B) = 0.537). Patients categorized as low responders are positively and significantly associated with the lesion to admission time (*p* < 0.001, Exp(B) = 1.015) and age (*p* < 0.001, Exp(B) = 1.038). The failure to respond on the SCIM scale is strongly associated with complications during the rehabilitation stay, which drastically increase the odds of being a non-responder (*p* = 0.008, Exp(B) = 16.834). Age is also positively associated (*p* = 0.009, Exp(B) = 1.055), whereas the year of admission presents a negative association (*p* = 0.034, Exp(B) = 0.877) (Table 1, Figure 2).

**Figure 3:**
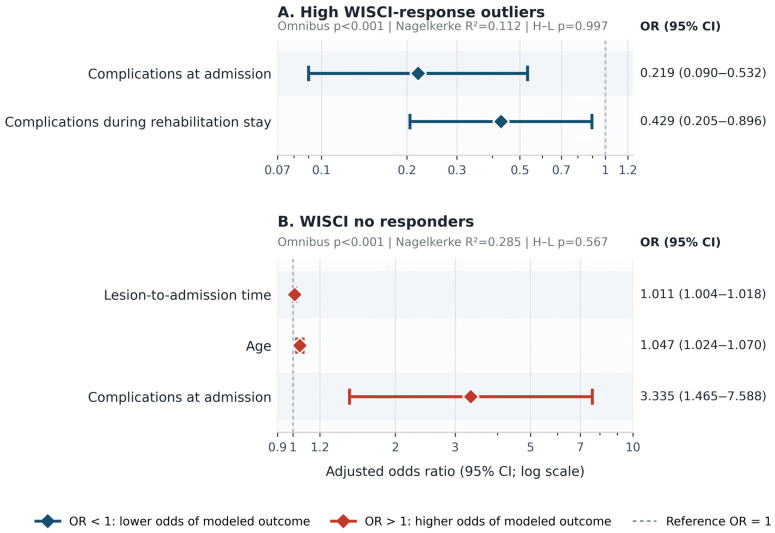
Adjusted odds ratios for predictors retained in the logistic-regression models for WISCI outcomes. Forest plots show adjusted odds ratios (ORs) and 95% confidence intervals (CIs) from forward stepwise logistic-regression models for: (**A**) WISCI high-response outliers, defined as patients with WISCI treatment effectiveness greater than the subgroup mean + 1 SD; and (**B**) WISCI non-responders, defined as patients with no WISCI improvement during rehabilitation. The dashed vertical line indicates OR = 1. ORs for age and lesion-to-admission time are expressed per one-year and per one-day increase, respectively. ORs for complications are expressed as presence versus absence. Values below 1 indicate lower odds of the modeled outcome, and values above 1 indicate higher odds of the modeled outcome. All models included 1059 patients. WISCI, Walking Index for Spinal Cord Injury; OR, odds ratio; CI, confidence interval; H–L, Hosmer–Lemeshow test.

As to walking capacity, 144 subjects were identified as WISCI outliers and 191 as WISCI non-responders. Being an outlier on the WISCI scale is negatively associated with clinical complications, specifically complications at admission (*p* = 0.001, Exp(B) = 0.219) and complications during the rehabilitation stay (*p* = 0.024, Exp(B) = 0.429). The odds of being a non-responder for mobility (WISCI) are positively associated with lesion to admission time (*p* = 0.002, Exp(B) = 1.011), age (*p* < 0.001, Exp(B) = 1.047), and complications at admission (*p* = 0.004, Exp(B) = 3.335). (Table 2, Figure 3).

**Figure 4:**
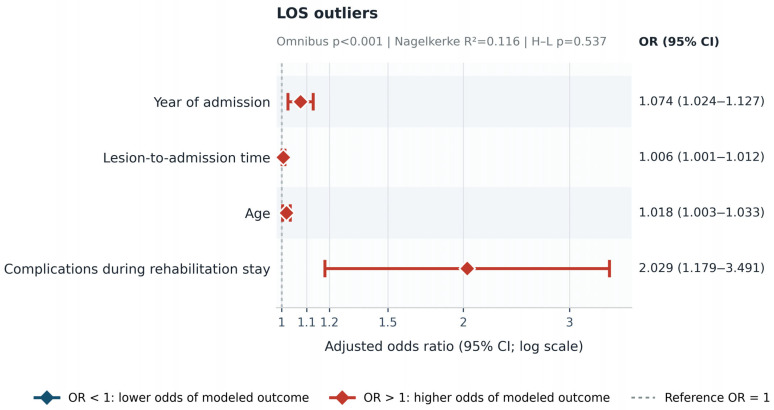
Adjusted odds ratios for predictors retained in the logistic-regression model for length-of-stay outliers. The forest plot shows adjusted odds ratios (ORs) and 95% confidence intervals (CIs) for being a length-of-stay (LOS) outlier, defined as a rehabilitation stay exceeding the subgroup mean + 1 SD. The dashed vertical line indicates OR = 1. ORs for continuous predictors are expressed per one-unit increase: one calendar year for year of admission, one day for lesion-to-admission time, and one year for age. The OR for complications during rehabilitation is expressed as presence versus absence. Values above 1 indicate higher odds of prolonged rehabilitation stay. The model included 1059 patients. LOS, length of stay; OR, odds ratio; CI, confidence interval; H–L, Hosmer–Lemeshow test.

With regard to LOS, 132 patients were classified as LOS outliers. Being a LOS outlier was positively and significantly associated with the year of admission (*p* = 0.003, Exp(B) = 1.074), lesion to admission time (*p* = 0.031, Exp(B) = 1.006), age (*p* = 0.022, Exp(B) = 1.018), and complications during rehabilitation stay (*p* = 0.011, Exp(B) = 2.029) (Table 3, Figure 4).

**Figure 5:**
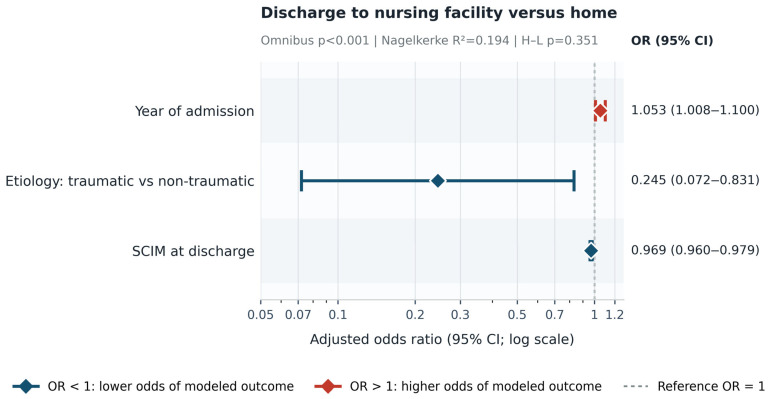
Adjusted odds ratios for predictors retained in the logistic-regression model for discharge destination. The forest plot shows adjusted odds ratios (ORs) and 95% confidence intervals (CIs) for discharge to a nursing facility versus discharge home. The dashed vertical line indicates OR = 1. ORs for year of admission and SCIM score at discharge are expressed per one-year and per one-point increase, respectively. The OR for etiology is expressed as traumatic versus non-traumatic spinal cord injury. Values above 1 indicate higher odds of nursing-facility discharge, whereas values below 1 indicate lower odds of nursing-facility discharge. The model included 1059 patients. SCIM, Spinal Cord Independence Measure; OR, odds ratio; CI, confidence interval; H–L, Hosmer–Lemeshow test.

At discharge, 913 patients returned home, whereas 146 were transferred to a nursing facility. Nursing home placement was positively associated with year of admission (*p* = 0.020, Exp(B) = 1.053), and negatively as-sociated with traumatic etiology (*p* = 0.024, Exp(B) = 0.245) and SCIM score at discharge (*p* < 0.001, Exp(B) = 0.969, 95% CI: 0.960–0.979), indicating that each additional SCIM point at discharge reduces the odds of nursing home placement by approximately 3%. (Table 4, Figure 5).

## 4. Discussion

Our results confirm the substantial variability in therapeutic response during spinal cord injury rehabilitation. Approximately 60% of the individuals in this cohort demonstrated a standard therapeutic response in activities of daily living (as measured by the SCIM) and mobility (as measured by the WISCI), while the remaining 40% showed either poor or excellent prognoses.

Moreover, our findings suggest that it is possible to identify, with reasonable confidence, factors associated with different therapeutic responses.

The Impact of Age on Rehabilitation Trajectories: The results demonstrate that older age is significantly associated with a reduced likelihood of robust functional recovery (being a SCIM or WISCI outlier) and an increased the risk of being a low/non-responder, as well as of experiencing a prolonged length of stay (LOS). To quantify these effects: each additional year of age was associated with a 4.3% reduction in the odds of high SCIM response (Exp(B) = 0.957), equivalent to approximately a 35% reduction per decade. For SCIM non-response, each additional year increased the odds by 5.5% (Exp(B) = 1.055), translating to nearly a threefold increase per decade in a 70-year-old compared with a 50-year-old. For LOS, the per-year increase was more modest (Exp(B) = 1.018), but it remained consistent across models, underscoring the systemic nature of age-related constraints on recovery. This is consistent with a recent study that found that increased age does not negatively affect neurologic recovery, including changes in total motor scores or sensory outcomes. However, older age is significantly associated with poorer functional and ambulation outcomes. Specifically, functional improvement declines by an estimated 4.3 Spinal Cord Independence Measure (SCIM) points for every decade of age [13]. Researchers also identified a critical age cutoff of 70 years, after which patients experience a pronounced reduction in functional recovery despite having similar neurologic recovery potential to younger patients. Older adults face challenges due to age-related factors that reduce their “vital capacity”, such as reduced muscle mass, decreased bone density, and the presence of comorbid conditions, all of which may hinder rehabilitation and limit functional gains [14]. Furthermore, it has been postulated that older subjects with SCI may encounter difficulties in translating their neurological recovery into functional improvement [14]. Altogether, these data indicate that rehabilitation programs must be specifically tailored for the aging SCI population, and rehabilitation interventions must be carefully calibrated to maximize functional gains while mitigating exhaustion and preventing injury.

Secondary Complications as Major Barriers to Recovery Clinical complications, particularly those developing during the rehabilitation stay, emerged as strongly associated with failing to respond functionally (SCIM/WISCI non-responders) and strongly increased the odds of an outlier LOS. The magnitude of the complication effect on SCIM non-response should be highlighted: patients who developed complications during their rehabilitation stay had approximately 17 times the odds of not showing any functional improvement (Exp(B) = 16.834; 95% CI: 2.087–135.806). The confidence interval is large, reflecting the small denominator of SCIM non-responders (*n* = 45), so the point estimate should be interpreted as an order-of-magnitude indicator rather than a precise value. Nonetheless, the direction and significance of this association are solid. Pre-existing complications had a significant impact on walking recovery: complications at admission reduced the odds of an exceptional WISCI response by about 78% (Exp(B) = 0.219). Previous studies corroborate that secondary complications like pressure ulcers, pneumonia, and urinary tract infections severely interrupt the rehabilitation process (Complications). For instance, respiratory issues and pressure ulcers can limit a patient’s mobility and their ability to participate in therapies, leading to missed therapy time and subsequent functional stagnation [15]. Additionally, complications such as pressure ulcers can induce a chronic inflammatory state characterized by anemia, low serum iron, hypoproteinemia, and hypoalbuminemia [16], all of which can significantly reduce functional potential [17]. These findings claim for an aggressive prevention of secondary medical complications as a cornerstone of effective SCI rehabilitation. To preserve functional potential and limit hospital stays, the rehabilitation team must implement preventative protocols—such as early mobilization, proper positioning, and rigorous skin assessments—as early as the acute care phase. Minimizing missed therapy time by proactively managing these preventable complications is essential for optimizing functional outcomes.

Process of Care: The Importance of Early Rehabilitation: The data shows that a longer lesion-to-admission time (the delay from acute injury to specialized rehabilitation admission) is positively associated with poorer functional (SCIM low and non-responders) and mobility (WISCI non-responders) outcomes and an extended LOS. Each additional day of delay was associated with a 1.5% increase in the odds of suboptimal SCIM response (Exp(B) = 1.015) and a 1.1% per day increase in the odds of WISCI non-response (Exp(B) = 1.011). While these per-day increments appear small, they accumulate: a 30-day delay—not uncommon in practice—corresponds to approximately 56% higher odds of low SCIM response and 39% higher odds of walking non-response, assuming log-linearity of the effect across the observed range. While some recent findings suggest that wait times might not severely impact long-term outcomes if a specialized acute rehabilitation program is administered during the wait [18], prolonged delays without targeted therapy are generally detrimental [19]. Delays in transferring patients to specialized inpatient rehabilitation often result in prolonged bed rest in acute settings, which accelerates skeletal and muscle loss, decreases functional capacity, and leads to severe deconditioning and possibly to other complications such as pressure ulcers [19]. Therefore, early transfer to a specialized SCI rehabilitation center is vital for maximizing recovery. When administrative or medical delays occur, it is imperative that a dedicated multidisciplinary rehabilitation process is initiated within the acute care setting. Early mobilization and targeted therapies during the waiting period help alleviate the negative impacts of immobility and better prepare the patient for intensive functional rehabilitation.

Shifting Demographics and Temporal Trends: The year of admission was negatively associated with the outcomes under examination, increasing the odds of an outlier LOS and impacting discharge destinations, and negatively correlating with SCIM outliers. This datum is counterintuitive, as one would expect that the advances in rehabilitation treatments (for example, by means of robotic treatment) could improve the recovery of subjects with SCI. However, it aligns with epidemiological data showing that the SCI population is progressively aging and presenting with greater medical complexity and comorbidity burdens over time [20]. As the demographic landscape of SCI shifts towards older and more medically complex individuals, episode costs and the required duration of rehabilitation naturally increase. Rehabilitation facilities must strategically adapt by allocating greater resources and developing highly specialized, injury-specific care pathways to manage this rising clinical complexity without compromising the quality of care.

Regarding the discharge destination, the results indicate that the year of admission is negatively associated with discharge destination, increasing the odds of being discharged to a nursing facility [21], while higher SCIM scores at discharge are associated with a more favorable discharge destination (e.g., returning home). A patient’s level of functional independence is a primary determinant of their ability to safely reintegrate into the community [22]. Conversely, discharge to an institution is frequently driven by a lack of physical support, inaccessible housing, and a high degree of dependence in daily activities [22]. The ultimate goal of intensive functional rehabilitation is successful community reintegration. By maximizing functional independence through rigorous, targeted therapy, rehabilitation professionals directly reduce the likelihood of institutionalization. This not only mitigates the long-term financial burden on the healthcare system but also vastly improves the psychosocial well-being, autonomy, and quality of life of the individual.

Notably, the predictors identified across models, namely age, complications, and lesion-to-admission delay, should not be considered in isolation. Older patients are inherently more susceptible to rehabilitation complications due to reduced skin integrity, impaired immunity, polypharmacy, and limited physiological reserve. A prolonged delay before specialized rehabilitation admission, in turn, promotes deconditioning and predisposes to the same complications. The co-occurrence of these three factors in the same patient would, under the assumption of multiplicative independence between predictors (i.e., the assumption implicit in the main-effects logistic regression model), produce substantially amplified risk. For illustrative purposes, a 70-year-old patient with complications at admission and a 30-day admission delay would face compounded odds of non-response that approximate the product of the individual odds ratios reported in each respective model. Formally testing these interactions was beyond the scope of this exploratory study and would require a prospective dataset with greater statistical power; nonetheless, the consistent co-occurrence of these variables across all models suggests a recognizable high-risk clinical phenotype. Clinically, older, late-admitted, and previously complicated subjects should be identified at the point of admission and represent a priority target for intensified prevention of secondary complications, accelerated admission pathways, and adjusted therapeutic goal setting. It is also worth noting that lesion level and AIS grade, which are commonly described as the primary determinants of SCI outcome, did not appear as independent regression predictors in any model. This does not mean they are irrelevant; rather, it reflects the design of this study. Because outlier status was defined as deviation from the mean within neurological subgroups already stratified by lesion level and AIS grade, these variables were effectively held constant by the classification procedure before regression was applied. The predictors identified here are therefore factors that differentiate outcomes among neurologically comparable patients, arguably the most clinically actionable dimension of the findings, since neurological severity is largely fixed at the time of admission, whereas age-related deconditioning risk, complication prevention, and admission timing can all be actively managed.

### Limitations

This study has several limitations.

Due to intensive care constraints, patients requiring mechanical ventilation are not admitted to the hospital where this study was conducted. The need for mechanical ventilation is clearly a factor that negatively affects outcomes and prolongs hospitalization in individuals with SCI.

Functional outcomes, LOS, and discharge destination are influenced by factors beyond clinical and demographic characteristics. Several “barriers to discharge”—including psychological [23] and social conditions, financial resources, housing availability, presence or absence of relatives or caregivers, and architectural barriers in the home [24]—were not considered due to the retrospective design of this study.

We acknowledge that the introduction of patients with non-traumatic SCI could introduce some heterogeneity because patients with non-traumatic lesions are different from those with traumatic SCI regarding age, level and completeness of the lesion. However, in previous works [25], it has been highlighted that, when the effect of these confounders is eliminated, the functional and neurological outcomes and the trajectory of recovery of the two groups are comparable. Etiology was entered as a candidate predictor in all models. It emerged as a significant independent predictor only for discharge destination, with traumatic SCI associated with lower odds of nursing home placement (Exp(B) = 0.245, *p* = 0.024), but did not reach statistical significance for SCIM or WISCI outcomes or for LOS, suggesting that its prognostic role in this cohort was limited to the social and logistic factors determining the ability to return home.

Complications were grouped for pragmatic reasons related to the retrospective design and to preserve model parsimony. This could introduce a bias because different complications may have a different impact on the outcomes. To address this concern, we performed an additional sensitivity analysis using pressure ulcers as the main complication variable, since pressure ulcers represented the most frequent complications in our cohort (Table S2, Supplemental Material). The overall pattern of results remained substantially unchanged.

The definition of LOS outliers based on mean + 1 SD is an operational one and may not be the only possible clinically meaningful threshold in a potentially skewed distribution. This threshold was chosen to remain consistent with previous rehabilitation “outlier” studies and with the exploratory aim of identifying patients at the extreme end of the LOS distribution.

The stepwise procedure that was used as an exploratory variable-selection approach in a large retrospective dataset may show model instability and the results need to be interpreted cautiously.

Finally, the study is a single-center one, and this may limit the generalizability of the results.

## Figures and Tables

**Table 1:** Logistic regression analysis for SCIM outliers and SCIM low or non-responders.

SCIM Outliers								
	B	E.S.	Wald	df	Sig.	Exp(B)	95% CI per EXP(B)	
							Inferior	Superior
Year of admission	−0.73	0.023	9.763	1	0.002	0.930	0.888	0.973
Age	−0.044	0.009	24.295	1	0.000	0.957	0.940	0.974
Complications at admission	−0.984	0.315	9.748	1	0.002	0.374	0.202	0.693
Complications during rehabilitation stay	−0.622	0.311	3.993	1	0.046	0.537	0.292	0.988
Costant	146.992	46.715	9.901	1	0.002	6.885 × 10^63^		
Test omnibus < 0.001; Nagelkerke Square R 0.248; Hosmer–Lemeshow Test 0.878 *n*= 1059								
**SCIM low responders**								
	B	E.S.	Wald	df	Sig.	Exp(B)	95% CI per EXP(B)	
							Inferior	Superior
Lesion to admission time	0.015	0.003	22.891	1	0.000	1.015	1.009	1.021
Age	0.037	0.009	15.579	1	0.000	1.038	1.019	1.057
Costant	−4.691	0.575	66.621	1	0.000	0.009		
Test omnibus < 0.001; Nagelkerke Square R 0.188; Hosmer–Lemeshow Test 0.302 *n* = 1059								
**SCIM non-responders**								
	B	E.S.	Wald	df	Sig.	Exp(B)	95% CI per EXP(B)	
							Inferior	Superior
Year of admission	−0.131	0.062	4.496	1	0.034	0.877	0.777	0.990
Age	0.053	0.020	6.810	1	0.009	1.055	1.013	1.098
Complications during rehabilitation stay	2.823	1.065	7.025	1	0.008	16.834	2.087	135.806
Costant	255,157	123.973	4236	1	0.040	6504 × 10^110^		
Test omnibus < 0.001; Nagelkerke Square R 0.284; Hosmer–Lemeshow Test 0.064 *n* = 1059								

**Table 2:** Logistic regression analysis for WISCI outliers and non-responders and WISCI.

WISCI Outliers									
	B	E.S.	Wald	df		Sig.	Exp(B)	95% CI per EXP(B)	
								Inferior	Superior
Complications at admission	−1.517	0.452	11.255	1		0.001	0.219	0.090	0.532
Complications during rehabilitation stay	−0.847	0.376	5.069	1		0.024	0.429	0.205	0.896
Costante	−1.326	0.182	53.203	1		0.000	0.266		
Test omnibus < 0.001; Nagelkerke Square R 0.112; Hosmer–Lemeshow Test 0.997 *n* = 1059									
**WISCI non-responders**									
	B	E.S.	Wald		df	Sig.	Exp(B)	95% CI per EXP(B)	
								Inferior	Superior
Lesion to admission time	0.011	0.004	9.174		1	0.002	1.011	1.004	1.018
Age	0.046	0.011	16.101		1	0.000	1.047	1.024	1.070
Complications at admission	1.204	0.419	8.243		1	0.004	3.335	1.465	7.588
Costant	−4.699	0.732	41.224		1	0.000	0.009		
Test omnibus < 0.001; Nagelkerke Square R 0.285; Hosmer–Lemeshow Test 0.567 *n* = 1059									

**Table 3:** Logistic regression analysis for LOS outliers.

LOS Outliers								
	B	E.S.	Wald	df	Sig.	Exp(B)	95% CI per EXP(B)	
							Inferior	Superior
Year of admission	0.072	0.024	8.697	1	0.003	1.074	1.024	1.127
Lesion to admission time	0.006	0.003	4.651	1	0.031	1.006	1.001	1.012
Age	0.018	0.008	5.240	1	0.022	1.018	1.003	1.033
Complications during rehabilitation stay	0.707	0.277	6.531	1	0.011	2.029	1.179	3.491
Costant	−147.343	48.952	9.060	1	0.003	0.000		
Test omnibus < 0.001; Nagelkerke Square R 0.116; Hosmer–Lemeshow Test 0.537 *n* = 1059								

**Table 4:** Logistic regression analysis for discharge destination.

Destination at Discharge								
	B	E.S.	Wald	df	Sig.	Exp(B)	95% CI per EXP(B)	
							Inferior	Superior
Year at admission	0.052	0.022	5.455	1	0.020	1.053	1.008	1.100
Etiology	−1.407	0.624	5.092	1	0.024	0.245	0.072	0.831
SCIM at discharge	−0.031	0.005	34.442	1	0.000	0.969	0.960	0.979
Costant	−103.085	44.651	5.330	1	0.021	0.000		
Test omnibus < 0.001; Nagelkerke Square R 0.194; Hosmer–Lemeshow Test 0.351 *n*= 1059								

## Data Availability

Data are available upon reasonable request to the corresponding author.

## Supplementary Materials

The following supporting information can be downloaded at https://www.mdpi.com/article/10.3390/jcm15114167/s1: Table S1: Collinearity statistics; Table S2: Logistic regression analysis for SCIM outliers with pressure ulcers instead of complications at admission.
