# Supplementary material for: Predicting Low and Non-Responders and Outliers in Patients with Spinal Cord Injury
Source: J Clin Med. 2026 May 28;15(11):4167. doi: 10.3390/jcm15114167 (PMC13257878; doi:10.3390/jcm15114167)
Supplement: Supplementary file 1 [file jcm-15-04167-s001.zip › jcm-4199085-supplementary.pdf]

Table S1. Collinearity statistics.

**Model for SCIM outliers**	Non standardized coefficients		Standardized coefficients	t	Sig.	Collinearity statistics	
	B	Standard deviation error	Beta			Tolerance	VIF
(Costant)	22.725	6.197		3.667	.000		
Year of admission	-.011	.003	-.171	-3.587	.000	.908	1.101
Lesion to admission time	-.001	.000	-.070	-1.367	.173	.791	1.264
Age	-.005	.001	-.248	-4.899	.000	.810	1.234
Etiology	.088	.063	.077	1.408	.160	.701	1.427
Associated lesions	-.063	.042	-.079	-1.497	.135	.740	1.352
Complication at admission	-.100	.041	-.122	-2.469	.014	.850	1.177
Complications during the rehabilitation stay	-.070	.039	-.085	-1.794	.074	.916	1.092
**Model for SCIM low respondes**	Non standardized coefficients		Standardized coefficients	t	Sig.	Collinearity statistics	
	B	Standard deviation error	Beta			Tolerance	VIF
(Costant)	9.772	5.134		1.903	.058		
Year of admission	-.005	.003	-.095	-1.931	.054	.908	1.101
Lesion to admission time	.002	.000	.224	4.287	.000	.797	1.254
Age	.003	.001	.192	3.709	.000	.816	1.226
Etiology	.007	.052	.007	.133	.894	.716	1.397
Associated lesions	-.031	.035	-.047	-.876	.382	.745	1.343
Complications at admission	.049	.034	.073	1.437	.152	.849	1.178
Complications during the rehabilitation stay	.056	.032	.084	1.717	.087	.918	1.090
**Model for SCIM no responders**	Non standardized coefficients		Standardized coefficients	t	Sig.	Collinearity statistics	
	B	Standard deviation error	Beta			Tolerance	VIF
(Costant)	5.069	2.661		1.905	.058		
Year of admission	-.003	.001	-.099	-1.955	.051	.907	1.102
Lesion to admission time	.000	.000	.097	1.806	.072	.799	1.252
Age	.002	.000	.169	3.183	.002	.821	1.219
Etiology	.073	.028	.152	2.663	.008	.711	1.406
Associated lesions	-.020	.018	-.061	-1.098	.273	.740	1.351
Complications at admission	.015	.018	.045	.867	.386	.850	1.177
Complications during the rehabilitation stay	.053	.017	.159	3.178	.002	.920	1.087
Model for WISCI outliers	Non standardized coefficients		Standardized coefficients	t	Sig.	Collinearity statistics	
	B	Standard deviation error	Beta			Tollerance	VIF

(Costant)	7.573	5.470		1.384	.167		
Year of admission	-.004	.003	-.069	-1.347	.179	.908	1.101
Lesion to admission time	.000	.000	-.048	-.875	.382	.791	1.264
Age	.000	.001	.009	.164	.870	.810	1.234
Etiology	.017	.055	.018	.315	.753	.701	1.427
Associated lesions	-.028	.037	-.043	-.755	.451	.740	1.352
Complications at admission	-.108	.036	-.158	-3.008	.003	.850	1.177
Complications during the rehabilitation stay	-.069	.034	-.101	-1.993	.047	.916	1.092
Model for WISCI no responders	Non standardized coefficients		Standardized coefficients	t	Sig.	Collinearity statistics	
	B	Standard deviation error	Beta			Tolerance	VIF
(Costant)	-1.335	9.368		-.143	.887		
Year of admission	.001	.005	.008	.125	.901	.874	1.144
Lesion to admission time	.002	.001	.198	2.802	.006	.763	1.311
Age	.005	.002	.234	3.278	.001	.744	1.344
Etiology	-.008	.073	-.008	-.109	.914	.701	1.427
Associated lesions	-.038	.060	-.045	-.638	.524	.758	1.320
Complications at admission	.187	.067	.186	2.777	.006	.851	1.175
Complications during the rehabilitation stay	.086	.056	.098	1.527	.128	.926	1.080
Model for LOS outliers	Non standardized coefficients		Standardized coefficients	t	Sig.	Collinearity statistics	
	B	Standard deviation error	Beta			Tolerance	VIF
(Costant)	-16.195	6.409		-2.527	.012		
Year of admission	.008	.003	.134	2.541	.011	.842	1.188
Lesion to admission time	.001	.000	.085	1.545	.123	.772	1.295
Age	.002	.001	.100	1.794	.074	.754	1.326
Etiology	-.037	.064	-.034	-.582	.561	.673	1.486
Associated lesions	.018	.042	.024	.420	.675	.731	1.369
Complications at admission	.032	.047	.041	.677	.499	.633	1.581
Complications during the rehabilitation stay	.093	.040	.121	2.309	.021	.853	1.172
Pressure ulcers at discharge	-.067	.056	-.072	-1.191	.234	.640	1.561
Micturition independence	.117	.076	.126	1.537	.125	.351	2.850
Faecal incontinence	-.045	.067	-.043	-.665	.506	.569	1.757
Independence in bowel management	-.060	.065	-.072	-.925	.356	.391	2.559
SCIM at discharge	-.002	.001	-.119	-1.465	.144	.356	2.810

Table S2. Logistic regression analysis for SCIM outliers with pressure ulcers instead of complications at admission.

SCIM outliers								
	B	E.S.	Wald	df	Sig.	Exp(B)	95% CI per EXP(B)	
							Inferior	Superior
Year of admission	-.078	.023	11.462	1	.001	.925	.884	.968
Age	-.045	.009	25.547	1	.000	.956	.940	.973
Pressure ulcers at admission	.875	.318	7.586	1	.006	2.398	1.287	4.469
Costant	155.972	46.091	11.452	1	.001	5.466E+067		
Test omnibus <0.001; Nagelkerke Square R 220; Hosmer-Lemeshow Test 0.784<br>*n*= 1059								
